# Evaluation of Paxillin Expression in Epithelial Dysplasia, Oral Squamous Cell Carcinoma, Lichen Planus with and without Dysplasia, and Hyperkeratosis: A Retrospective Cross-Sectional Study

**DOI:** 10.3390/diagnostics13152476

**Published:** 2023-07-25

**Authors:** Seyedeh Sara Aghili, Razieh Zare, Alireza Jahangirnia

**Affiliations:** 1 Student Research Committee, School of Dentistry, Shiraz University of Medical Sciences, Shiraz 71348-53734, Iran; saraaghili95@gmail.com; 2 Department of Oral and Maxillofacial Pathology, School of Dentistry, Shiraz University of Medical Sciences, Shiraz 71348-53734, Iran; 3 Independent Researcher, Tehran 19589-13863, Iran

**Keywords:** dysplasia, squamous cell carcinoma, lichen planus, paxillin, carcinomatosis

## Abstract

Background: Paxillin is a cytoskeletal protein involved in the pathogenesis of several types of cancers. However, the roles of paxillin in epithelial dysplasia (ED), oral squamous cell carcinoma (OSCC), oral lichen planus with dysplasia (OLPD), hyperkeratosis (HK), and oral lichen planus (OLP) have remained unnoticed in the literature. This study aimed to evaluate its attainable functions in the pathogenesis and malignant transformation of potentially malignant oral epithelium and benign lesions. Methods: In this retrospective cross-sectional study, paxillin expression was investigated in 99 tissue samples, including 18 cases of OSCC, 21 ED, 23 OLP, 21 OLPD, and 16 cases of HK. The tissue sections also underwent immunohistochemical paxillin staining using 3,3-diaminobenzidine (DAB) chromogen. The intensity, location, and percentage of staining were examined across all groups. Data were analyzed using the Shapiro–Wilk test, ANOVA, Pearson chi-square, Kruskal–Wallis, and Dunn’s post hoc test. Results: The cytoplasmic percentage and intensity staining of Paxillin expression were evident in the central/suprabasal and basal/peripheral layers of all the obtained samples. The final staining score was significantly higher in OSCC and dysplasia compared to HK and OLP (*p* = 0.004). It was found that paxillin expression is associated with the grade of dysplastic samples (*p* < 0.001). Conclusion: The present study provides evidence that paxillin may be involved in the pathogenesis of OSCC and the development and progression of dysplastic tissue, since the paxillin expression was higher than that of HK and OLP.

## 1. Introduction

Oral squamous cell carcinoma (OSCC), the most prevalent malignancy in the head and neck squamous cell carcinoma category, was reported to rank 11th and 18th globally in 2012 and 2018 [1,2]. OSCC is characterized by high recurrence, facilitated metastasis, and local invasion [3,4]. Multimodality treatments, such as surgery, radiation, and/or chemotherapy, are required to manage OSCC [1,5]. Despite numerous improvements in OSCC treatment, the disease’s survival rate is still low [6,7,8]. Due to the poor survival rate, researchers are now studying the molecular processes involved in carcinogenesis. Carcinogenesis is a multi-step process characterized by the development of various mutations and anomalies in the epigenome that affect the expression of several genes with a wide range of functions [9,10,11]. As a result of enhanced proliferation, decreased apoptosis, and increased tumor cell motility, there is initially a loss of control over the cell cycle, which results in invasion and metastasis. Neoplastic epithelial cells can penetrate the basement membrane, invade the tissues beneath it, and reach nearby lymph nodes [12]. Oral potentially malignant disorders (OPMDs), such as oral leukoplakia (OLK), erythroplakia, or lichen planus, as well as histologically normal oral mucosa, may both develop into OSCC. This process begins at the normal epithelium and progresses through hyperplasia to dysplasia before ending in an invasive carcinoma [13]. The two most significant recognized risk factors for developing oral squamous cell carcinoma are likely tobacco use and alcohol consumption [14]. Moreover, age, gender, race, other oral habits (chewing tobacco and snuff), and viruses are other potential OSCC risk factors [15].

Dysplasia refers to abnormal cell growth. The oral epithelial dysplasia (ED) grade helps determine whether a lesion could likely progress to malignancy. The current gold standard for predicting the malignant transformation of OPMDs is the histopathologic evaluation for the presence of oral epithelial dysplasia [16,17]. The existence of epithelial dysplasia is a sign of OPMDs’ propensity for malignancy, and as the grades of epithelial dysplasia rise, so does the possibility that these lesions may develop into carcinoma [18,19]. Epithelial dysplasia is an entity with some histopathologic abnormalities detected when the morphology of epithelial cells becomes atypical, which is graded based on its severity as mild, moderate, or severe [20]. The risk of oral epithelial dysplasia may increase with tobacco smoking and alcohol consumption [21].

One of the chronic inflammatory disorders of the oral mucosa is oral lichen planus (OLP), which the WHO classifies as a potentially malignant lesion [22,23,24,25]. It is also a chronic auto-immune disorder mediated by T-cells in which cytotoxic T-cells (CD8+) target oral epithelial cells and trigger apoptosis [26]. The possibility of OLP developing cancer is still debatable and controversial; however, the risk was estimated to be within 0.5–12%. Furthermore, OLP’s malignant transformation mechanism is still unknown [27,28], but it is generally thought to be a multifactorial process with a variety of triggers, including mechanical, electrochemical, trauma, psychological, infectious, malnutrition, stress, and overwork, as well as mucous-exciting factors, allergies, endocrine disorders, disorders of the salivary glands, genetic susceptibility, and immunological illnesses [29,30,31]. Therefore, it should be regarded as a potentially cancerous lesion, and periodic follow-ups should be encouraged in all the affected patients [26].

Paxillin (PXN), first discovered in 1990, is a 68-kDa, adhesion-associated, and phosphotyrosine-containing protein that recruits signaling molecules into a complex. Structurally, it is considered as composed content, mediating protein-protein interactions. The domain-binding site’s contents act as sites for the cell cytoskeleton, tyrosine kinases, GTPase activating proteins, and other adaptor proteins that impress additional enzymes into the complex with paxillin [32]. Accordingly, paxillin plays pivotal functions in cell migration, focal adhesions, development, regulation of cell movement, immune response, epithelial morphogenesis, and embryonic development. In particular, paxillin imparts an undeniable part of pathological conditions, including oxidative stress, inflammation, and cancer development and metastasis [33]. The over-expression of paxillin has been reported with both the tumor progression and metastasis in numerous types of cancer, such as human esophageal squamous cell carcinoma [34], salivary adenoid cystic carcinoma [35], colorectal cancer [36], laryngeal carcinoma [37], prostate carcinoma [38], lung carcinoma [39], human breast cancer [40], and Kaposi’s carcinoma [41], in which the overexpression is associated with lymph node metastases, advanced stage, decreased survival, and poor prognosis. To the best of our knowledge, few studies have previously evaluated the role of paxillin as a prognostic and predictive marker in OSCC, and in particular oral potentially malignant lesions, such as OLP, leukoplakia, oral submucous fibrosis, and oral epithelial dysplasia [25,42,43,44,45,46]. Moreover, since paxillin is accepted as an oncogene in oral carcinogenesis, in the current study, we aimed to evaluate the expression of paxillin in different grades of OSCC, oral lichen planus, oral epithelial dysplasia, and hyperkeratosis, exploring its eventual roles as a prognostic and predictive marker, and evaluating the possible malignant transformation of oral lesions.

## 2. Materials and Methods

### 2.1. Patients and Tissue Selection

In this cross-sectional study, 99 patients diagnosed with oral hyperkeratosis (HK) in 16 cases, OLP in 23, ED in 21, OSCC in 18, and lichen planus with dysplasia (LPD) in the rest of them (21 cases) were selected based on the clinically archived tissues and their medical records in the Oral and Maxillofacial Pathology Department of Shiraz Dental School, Shiraz, Iran, between 2011 and 2018. Histopathological criteria adopted for definite diagnosis of OLP were: (1) the presence of a well-defined band-like mainly lymphocytic infiltrate that is confined to the connective tissue’s superficial part; (2) signs of keratinocyte apoptosis and vacuolar degeneration of the basal and/or suprabasal cell layers; and (3) epithelial thinning and sometimes ulceration in the atrophic type [47,48]. In addition, HK histologic characteristics were defined as (1) a significantly thickened granular zone with a higher percentage of small and large, irregularly shaped basophilic keratohyalin-like bodies, and amorphous eosinophilic trichohyalin-like bodies; (2) reticulate, lightly staining, amphophilic material forming indistinct cellular boundaries; and (3) a variety of sizes of clear spaces around nuclei in the stratum spinosum and the stratum granulosum [49].

All the obtained samples were from a definitive diagnosis, and the related hematoxylin-eosin (H&E) slides were re-evaluated to confirm this diagnosis. After that, erosive and ulcerative OLP samples with no definitive diagnosis and defective epithelial components were excluded. The patient’s medical records with OLP diagnosis were evaluated, and if the clinical and histopathologic diagnoses were OLP, their slides would be used. The patients with lichenoid reaction diagnosis or no bilateral lesions were excluded.

Moreover, poorly stained samples and small-sized ones that lead to a wrong diagnosis were excluded. Demographic data of the included cases, including age, gender, and the histological characteristics of each lesion, were also obtained from the patient’s medical records. There was no record of patients’ oral habits (alcohol, tobacco, betel quid, etc.) available. Subsequently, the histopathologic grading of the dysplastic and tumoral specimens was performed to assess the probability of the malignant transformation.

The minimum required sample size was calculated to be at least 80 participants (16 in each group) using GPower version 3.1.9.2, considering type one and type two errors to be 0.05 and 0.2, with an effect size of 0.4 based on Cohen’s criteria.

### 2.2. Ethical Approval

The process of decision making on the use of human tissue specimens is usually the responsibility of the Institutional Review Board (IRB), which is also known as the Institutional Ethics Committee (IEC) of Shiraz University of Medical Sciences (SUMS). The Ethics Committee of Shiraz University of Medical Sciences, Shiraz, Iran, approved the study design (IR.SUMS.DENTAL.REC.1398.134). The current study was performed in terms of all the institutional ethical guidelines. The Ethics Committee/Institutional Review Board of SUMS waived the need for informed consent since the research was conducted on tissues already diagnosed by pathologists, which did not cause any serious physical harm to human subjects.

### 2.3. Immunohistochemistry

The detection of paxillin expression was performed with immunohistochemical staining in terms of the standard protocols. Accordingly, this was followed by serial cutting of formalin-fixed and paraffin-embedded tissue blocks at a thickness of 4 μm. The xylene was used for the deparaffinized sections, graded alcohol series for rehydration, and 3% hydrogen peroxide in methanol, to block the endogenous peroxidase activity. After that, the sections were rinsed with phosphate-buffered saline and then subjected to microwave antigen retrieval in 0.01 mol/L sodium citrate buffer (pH 6.0). Rabbit anti-paxillin antibody (1:500, Abcam Corporation, ab 32084, Waltham, MA, USA) was used as the primary antibody, followed by horseradish peroxidase-streptavidin complex. Finally, adding 3,3-diaminobenzidine (DAB) chromogen visualized the labeled antibody, and all the sections were then counterstained with hematoxylin, which is generally used as background staining. The current study used colorectal cancer cells as the positive control [50], and the internal positive control included endothelial cells. The negative control was obtained by replacing the primary antibody with a phosphate-buffered saline solution (PBS), leading to the omission of the primary antibody.

The cytoplasmic percentage of positive cells was determined by examining 10^3^ cell populations/each lesion at ×400 magnification. The percentage and intensity of immunoreactivity were assessed to quantify the paxillin expression by modifying Jaafari’s method [51]. The percentage of staining was graded regarding the percentage of cells with a positive immunoreactivity in the outside cells of tumoral nests or the basal layer of both epithelium and middle cells of tumoral nests or suprabasal layer as follows: 0 = less than 10%; 1 = weak (10–25%); 2 = moderate (26–50%); 3 = strong (51–75%); and 4 = very strong (76–100). The overall staining intensity was assessed in contrast with the stained vessels, and scores ranged from 0 to 3 and were graded as follows: 1 = weak, 2 = moderate, and 3 = strong. The mean total score was obtained (ranging from 0 to 12) by multiplying the percentage of each sample in each locality with its overall staining intensity. Based on the immunohistochemical results, the patients who scored ≤6 were regarded as the low-level paxillin expression, and those who scored >6 were the high-level paxillin expression [51]. Some variations in the intensity of staining in each layer were observed between the same samples. These were according to the time tissue aimed to reach the lab for fixation.

### 2.4. Statistical Analysis

Data were analyzed using SPSS software (IBM SPSS Statistics version 24, IBM SPSS Inc., Chicago, IL, USA) at a 0.05 significance level. Quantitative and qualitative data were presented by mean ± standard deviation and number (percent), respectively. The Shapiro–Wilk test was used for the assessment of the normality assumption. Pearson chi-square, one-way analysis of variance (ANOVA), or Kruskal–Wallis followed by Dunn’s post hoc test was performed for the pairwise comparison of the groups regarding demographic characteristics or the study outcomes.

## 3. Results

In the present study, 99 patients were eligible to participate, including 57 women and 42 men, with a mean age of 50.7 years old, ranging from 15 to 83 years old. Baseline data, such as age and gender, SCC, and Dysplasia grade, are illustrated in Table 1.

**Figure 1:**
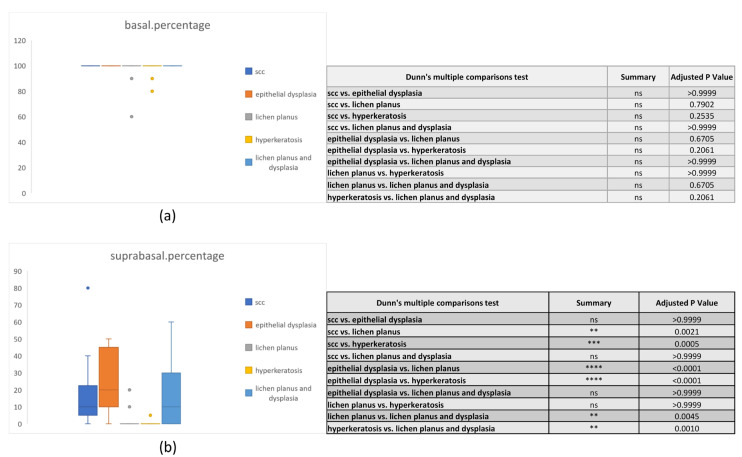
Paxillin overall percentage in the basal (**a**) and suprabasal (**b**) layers of all study groups. (ns: Non-significant, ** *p* < 0.01, *** *p* < 0.001, **** *p* < 0.0001).

**Figure 2:**
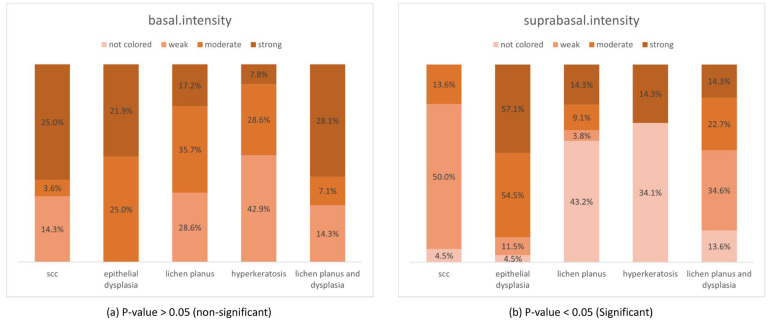
Paxillin overall intensity in the basal (**a**) and suprabasal (**b**) layers of all study groups.

The basal percentage was not significant between any of the study groups. However, the study groups saw significant pairwise differences in suprabasal percentage (Figure 1). In addition, the overall staining intensity expression varied significantly in the basal layers but not in the suprabasal layers among the study groups (Figure 2).

**Figure 3:**
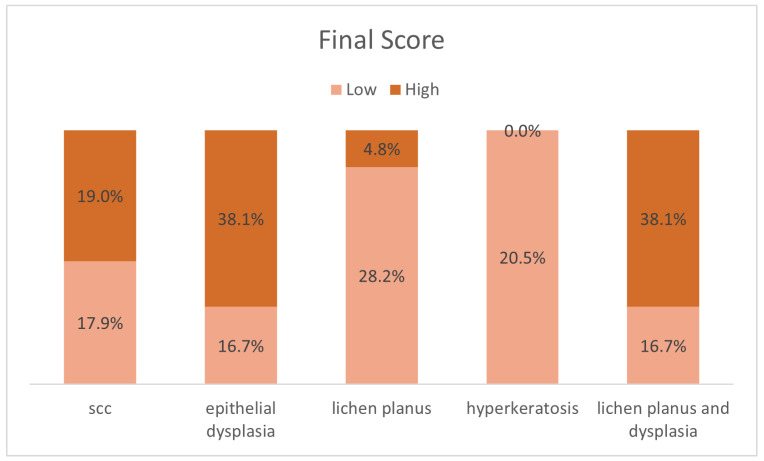
Paxillin’s mean final scores in all study groups.

The mean final scores (categorized from low to high) of the paxillin expression among all the focus groups (including SCC, ED, OLP, HK, and OLPD) were statistically significant (*p* = 0.004) (Figure 3).

**Figure 4:**
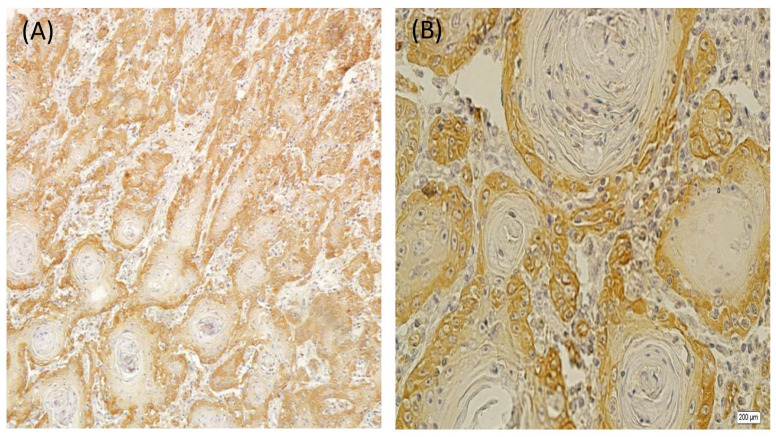
Cytoplasmic immunohistochemical staining of paxillin in OSCC: peripheral cells staining but no staining in central cells and keratin pearls ((**A**): ×100) ((**B**): ×400).

In the OSCC samples, paxillin overexpression in the basal layer was almost found in all cases (96%), but only 40% of the cases showed paxillin overexpression in the suprabasal layer (Figure 4). The overall staining intensity in the basal layer was vigorous, and that in the suprabasal layer was weak. Furthermore, more than half of the samples had a high final score. The central area and cells of large sheets around the keratin pearls and muscles were not stained. In contrast, endothelial cells, small nests, and cords were entirely stained. The salivary gland duct cells observed in one of the samples were stained, while acinar cells were left unstained. No significant association was detected between paxillin expression and the OSCC histological grade (*p* > 0.05).

**Figure 5:**
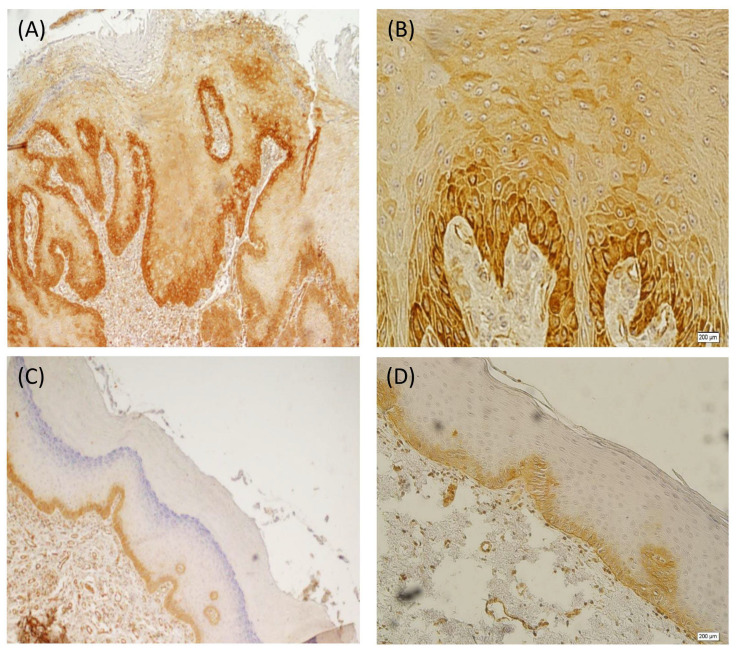
Cytoplasmic immunohistochemical staining of paxillin in ED: positive staining in basal and suprabasal layers ((**A**): ×200) ((**B**): ×400). Cytoplasmic immunohistochemical staining of paxillin in the basal layer of HK ((**C**): ×40) ((**D**): ×200).

In the ED group, 100% of the cases were stained for paxillin in the basal layers; however, 48% were stained for paxillin in the suprabasal layers (Figure 5A,B). The overall staining intensity was moderate in the basal layer and strong in the suprabasal layer. Moreover, high-grade dysplastic lesions showed an elevated intensity level of paxillin expression in the suprabasal layer (*p* = 0.013). Of note, the paxillin overexpression increased with the progression of dysplastic grade (*p* < 0.001). Furthermore, 38.1% of them had a high final score. Notably, most of the HK samples had paxillin overexpression in the basal layers (95%), and those with suprabasal layer staining of paxillin had the lowest rate (3%) (Figure 5C,D). Moreover, all these samples (100%) had a low final score. At this stage, superficial keratins as well as ortho-keratinized and para-keratinized ones were left unstained.

**Figure 6:**
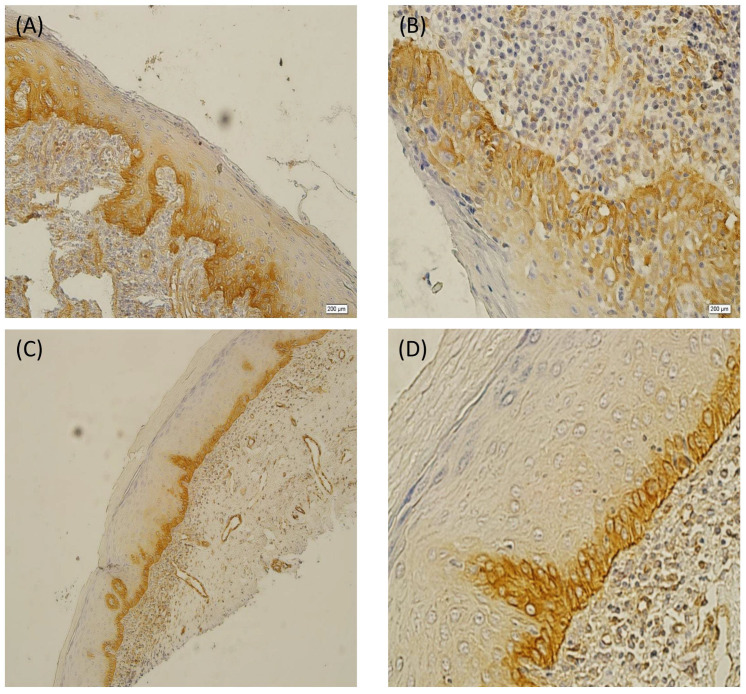
Cytoplasmic immunohistochemical staining of paxillin in OLPD: positive staining in basal and suprabasal layer ((**A**): ×200) ((**B**): ×400). Cytoplasmic immunohistochemical staining of paxillin in the basal layer of OLP ((**C**): ×100) ((**D**): ×400).

In the LPD cases, 58% of the obtained samples showed paxillin overexpression in the suprabasal layer (Figure 6A,B). Their basal cell layers’ overall staining intensity level was also strong and weak in the suprabasal cell layers. In addition, 52.4% of them had a high final score. Only 8% of the OLP cases showed paxillin overexpression in the suprabasal layer (Figure 6C,D). The overall staining intensity in the basal layer was moderate, with three cases showing high staining in the suprabasal layer. Furthermore, nearly all of the samples had a low final score. Inflammatory cells beneath the epithelial layer were stained as well.

In all the samples of all the study groups, the basal/peripheral layers demonstrated more prominent staining than the suprabasal layer. On the other hand, the highest staining percentage of the suprabasal layer belonged to the ED samples, and the lowest belonged to the HK samples.

In both the malignant (OSCC) and dysplasia groups (ED, LPD), the overall staining intensity of paxillin in basal and suprabasal layers and the mean final score were higher than those of HK and OLP (*p* > 0.005, *p* < 0.005, *p* = 0.004), respectively.

The vessels were consistently applied as an internal control of antibody activity and staining intensity. Notably, stroma staining was bound to endothelial cells, and no staining was seen in stromal fibroblasts.

## 4. Discussion

Paxillin plays a pivotal role in cell morphologic changes, cell motility, adhesion, migration, and cell signaling [52,53]. In addition, cell adhesion molecules play a role in adhesion and function as tumor suppressors [54]. Disorganized cell-cell or cell-extracellular matrix (ECM) attachment can significantly be provided towards unmanaged cell proliferation and progressive deformity of normal tissue construction [55]. Therefore, its expression in benign lesions is an essential factor that should be explored. Russo et al. discussed the epigenetic changes involved in the carcinogenesis process of oral and oropharyngeal cancers, and they showed how these epigenetic changes may be beneficial as potential predictive biomarkers [56].

The present study found higher paxillin expression in patients with malignancy (OSCC) and dysplasia (ED, LPD) compared to HK and OLP. The paxillin expression was also more observed in the tissues with dysplastic changes than in the non-dysplastic tissues. Hence, it can be suggested that paxillin over-expression might play an essential role in developing dysplastic tissues into malignancy. These outcomes align with Alam’s and Mackinnon’s findings [46,57]. These studies discovered that paxillin is overexpressed in high-risk patients’ dysplastic lesions, carcinoma, and potentially malignant areas of hyperplasia, squamous, and goblet cell metaplasia [46,57]. Some investigations have also reported a high paxillin expression in some types of malignancies, such as oral squamous cell carcinoma [45], colorectal cancer [36], salivary adenoid cystic carcinoma [35], and esophageal squamous cell carcinoma [34]. Furthermore, paxillin level was highly linked with progression and malignant metastasis [33].

Interestingly, previous studies on urothelial and lung carcinoma claimed that down-regulation in the paxillin expression might affect cell motility and promote invasiveness [50,58]. Additionally, differences in cell/tumor type, extracellular matrix, and engagement of different signaling molecules are thought to be the underlying causes of the discrepancies among the results of various studies performed in this field [50]. The scoring technique and kind of antibodies adapted were also ascribed to different outcomes of these studies [51]. Accordingly, we used quantitative, semi-quantitative, and staining intensity in the present study to find the study’s association. All these criteria proved the results’ analogy. In normal oral tissue, paxillin has been seldom described in humans previously, and few reports are available similar to the results we have reported here. In this study, we attempted to explain the paxillin expression with a broader range of functional alterations, with all benign cases being positive. In contrast with another report, Mackinnon and Li-B [34,57] have suggested that baseline levels of paxillin expression in normal tissues are tissue dependent [57].

OLP is considered a potentially malignant inflammatory lesion with a malignant transformation rate of up to 12% [27]. No study has addressed the paxillin expression in OLP up to now. In line with the paxillin expression in other lesions, our study also reported paxillin expression in OLP. In the current study, the OLP samples showed higher paxillin expression in the suprabasal layer than in HK. Multiple studies are available focusing on oncogenes and tumor suppressor genes’ expressions that connect with paxillin and OLP. In this regard, Kornberg et al. [59] have announced that FAK expression increases in oral cancer. The paxillin expression also increases the phosphorylation of FAK, which causes a reduction in P53 [60], and this may play a role in cancer development and progression [60]. In another study, Mattila et al. revealed caspase cascade pathways in patients with OLP [61], which are connected with paxillin [62]. It has been shown that inflammatory signaling of caspases increases paxillin breakdown, consequently diminishing paxillin expression [62]. OLP is assumed to be an auto-immune disease. Based on its molecular processes, there is a deficit of transforming growth factor-ß1 (TGF-ß1), which makes weak antigen-specific immunosuppression in OLP [63]. In addition, prior research generally confirmed that TGF-ß modulates the paxillin expression by interacting with its receptor; therefore, it may play a role in the OLP pathogenesis [64]. These findings may, to some extent, describe the paxillin expression reduction in our OLP samples.

Notably, the specimens with OLP manifested a lower paxillin expression than the ED and OSCC samples (*p* = 0.001, *p* = 0.001), respectively. The underlying cause of this finding may be that paxillin plays a role in pathological conditions, such as inflammatory processes [33]. Correspondingly, these outcomes could be known as the indicators of the act of paxillin in OLP pathogenesis. Since paxillin is involved in the malignant transformation process, we can relate its lower expression in OLP, rather than in OSCC, to its potentially malignant state. Consequently, greater paxillin expression was expected by proceeding to a malignant condition [33]. However, the exact paxillin function in malignancy is not clarified yet and is still under debate [26]. Lower paxillin expression may also be associated with less potential for malignant transformation of OLP than ED. On the other hand, Muñoz et al., in their study, have found that it takes OLP lesions 5.5 years to progress into an OSCC. Furthermore, the study has shown that patients with OSCC developed on a previous OLP lesion have a higher recurrence rate when compared with primary OSCC [65]. Recent research has also shown that a high suprabasal intensity level of paxillin in ED, rather than in OSCC, may be associated with early appearances in oral carcinogenesis since the frequency of paxillin suprabasal intensity in invasive types of cancer was estimated to be lower than the frequency of these in potentially cancerous lesions (*p* = 0.001) [55]. This outcome validated the result reported in the study by Madan et al. [58].

In the present study, the level of paxillin increased with the grade of the samples; however, the correlation was not statistically significant, which may be due to the usage of a small number of samples in each group, which is consistent with the investigation of Kandelman et al. [55]. Several studies have demonstrated that paxillin expression is associated with tumor grade in some kinds of tumors, such as in OSCC [45], benign and malignant salivary gland tumors [66], and colorectal cancer [36].

Additionally, in the dysplastic groups, paxillin was highly expressed at the advanced dysplastic stages. It is unclear whether paxillin is associated with invasion since it manages focal adhesion kinase (FAK) duty [67], which is known as a marker of malignant transformation rather than its invasion [68].

Similar to the findings of Kandelman et al. and Mackinnon et al. [55,57], our study showed that the basal layer paxillin expression was more prominent than the suprabasal. Small nests and cords in the OSCC indicated greater paxillin expression than the larger sheets, confirming greater paxillin expression in cells of tumors with a basal-like phenotype. Instead, the overexpression of paxillin perhaps expresses a duty related to the proliferation process. The respective elevation in the paxillin expression in both the basal and suprabasal layers and along the leading edge of offensive lesions substantiates this. Unfortunately, the OSCC samples were insufficient to assess the paxillin expression and type of invasion in these tumors. We theorized that when histological evidence of neoplasia initially appears, paxillin becomes overexpressed in benign lesions. According to current models, oral cancer progresses across a continuum of histological abnormalities in the normal epithelium as it advances through grades. These modifications are caused by an underlying buildup of molecular changes, which ultimately result in micro-invasiveness [57].

Larger samples may be able to show the malignant potential of OLP. However, OLP with dysplastic changes is not a typical lesion, and section preparation for this goal may be difficult.

Numerous investigations on the function of paxillin in cells using various methods have shown the same results, indicating that paxillin regulates cell spreading and motility. Nevertheless, this depends on the context. Paxillin could therefore be considered a helpful biomarker for both treatment and prognosis. However, additional research involving a sizable sample size, prospective long-term follow-ups, multiple geographic locations, local lesion biopsies, as well as other molecular analytical techniques may be required to draw a conclusive conclusion regarding the relationship between paxillin and the precise pathogenesis of potentially cancerous disorders and OSCC.

## 5. Conclusions

In conclusion, the present study provides evidence that paxillin may be involved in the pathogenesis of OSCC and the development and progression of dysplastic tissue since the overall staining intensity of paxillin in both the OSCC and dysplastic groups was higher than that of HK and OLP. In all the groups, the basal layers of the lesion demonstrated more prominent staining intensity than that of suprabasal layers, which may refer to the proliferative activity of paxillin. So, paxillin may be a prognostic marker in dysplastic lesions.

## Figures and Tables

**Table 1:** Baseline data of all study groups.

Variable		Total *N* = 99		SCC *N* = 18		Epithelial Dysplasia *N* = 21		Lichen Planus *N* = 23		Hyperkeratosis *N* = 16		Lichen Planus and Dysplasia *N* = 21		*p*-Value
Age		50.70	14.15	53.17	15.20	51.90	15.44	48.22	11.58	47.31	12.40	52.67	15.99	0.612
GENDER	F	57	57.6%	10	17.5%	12	21.1%	16	28.1%	8	14.0%	11	19.3%	0.737
	M	42	42.4%	8	19.0%	9	21.4%	7	16.7%	8	19.0%	10	23.8%	
SCC grade	Well-differentiated	13	72.2%	13	100.0%	0	0.0%	0	0.0%	0	0.0%	0	0.0%	NA
	Moderate	4	22.2%	4	100.0%	0	0.0%	0	0.0%	0	0.0%	0	0.0%	
	Severe	1	5.6%	1	100.0%	0	0.0%	0	0.0%	0	0.0%	0	0.0%	
Dysplasia grade	Mild	18	42.9%	0	0.0%	5	27.8%	0	0.0%	0	0.0%	13	72.2%	<0.001
	Moderate	8	19.0%	0	0.0%	8	100.0%	0	0.0%	0	0.0%	0	0.0%	
	Mild-moderate	8	19.0%	0	0.0%	0	0.0%	0	0.0%	0	0.0%	8	100.0%	
	Moderate-severe	3	7.1%	0	0.0%	3	100.0%	0	0.0%	0	0.0%	0	0.0%	
	Severe	5	11.9%	0	0.0%	5	100.0%	0	0.0%	0	0.0%	0	0.0%	

## Data Availability

The datasets used and/or analyzed during the current study are available from the corresponding author upon reasonable request.
